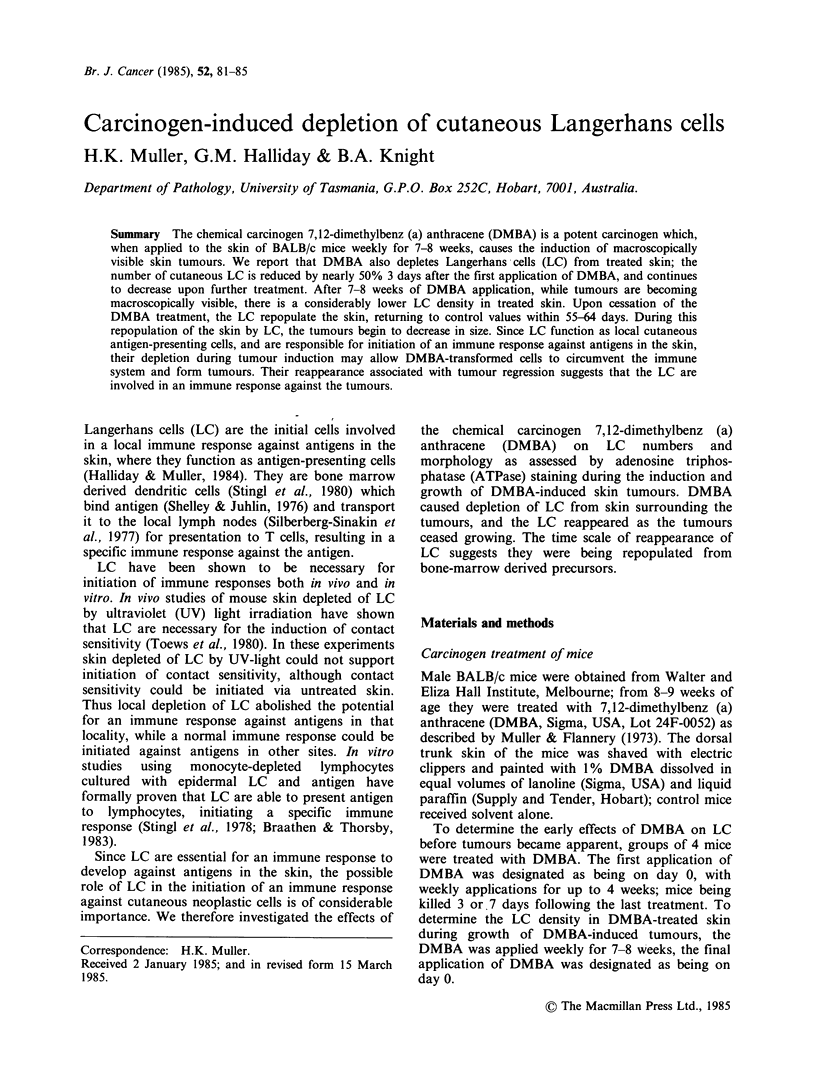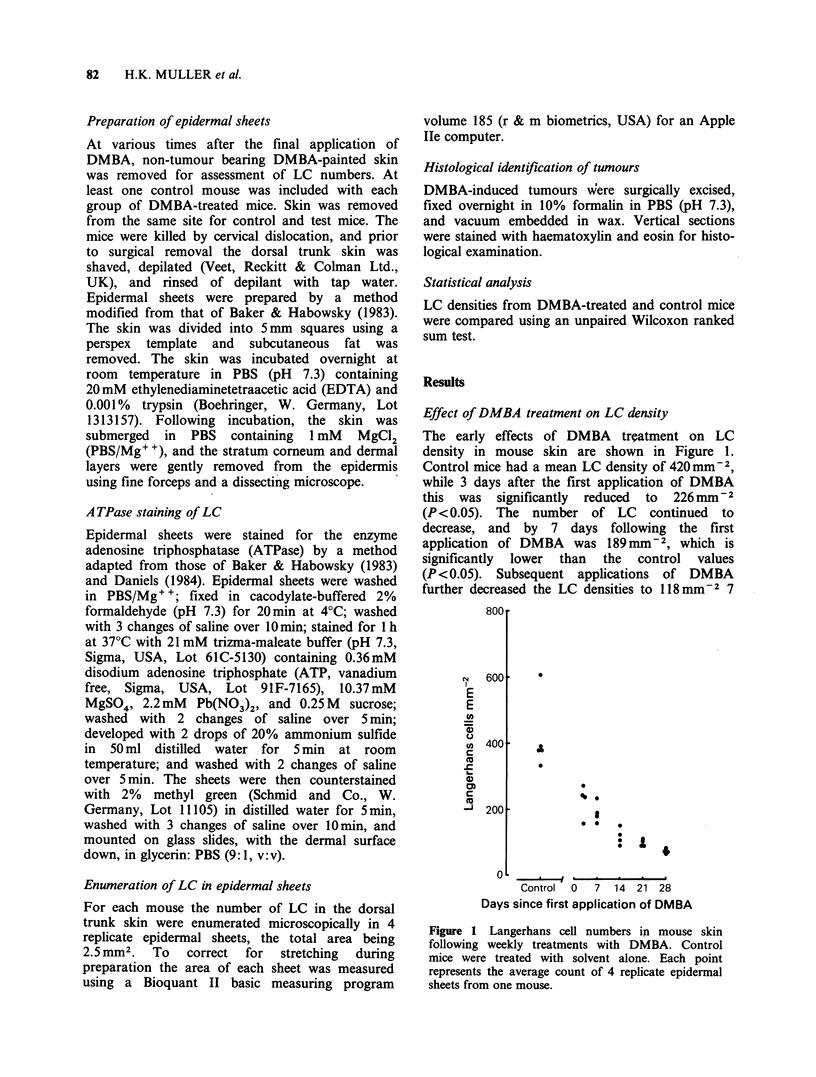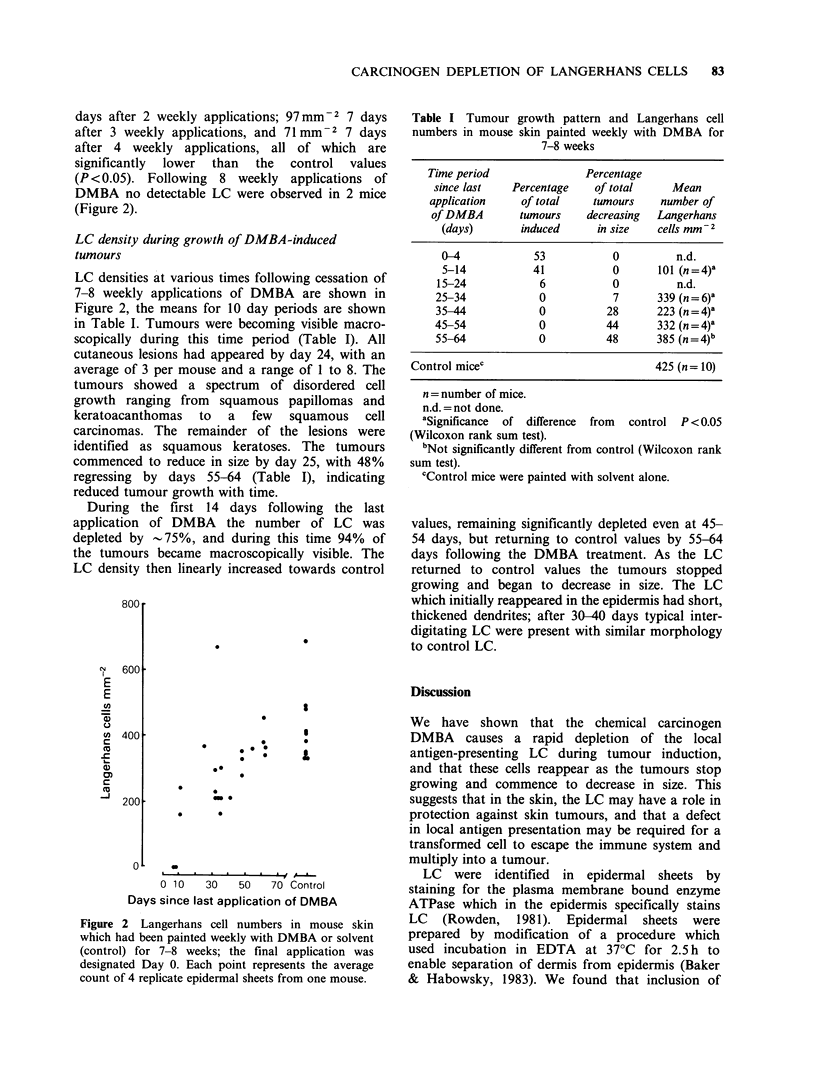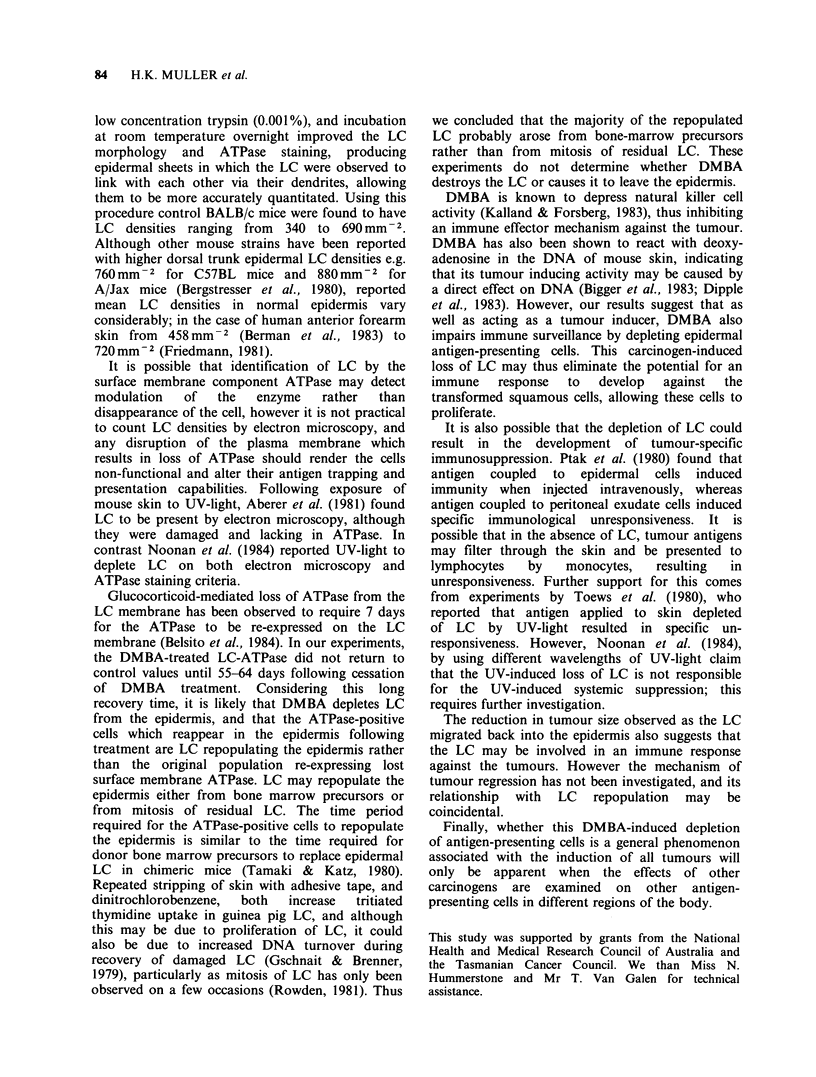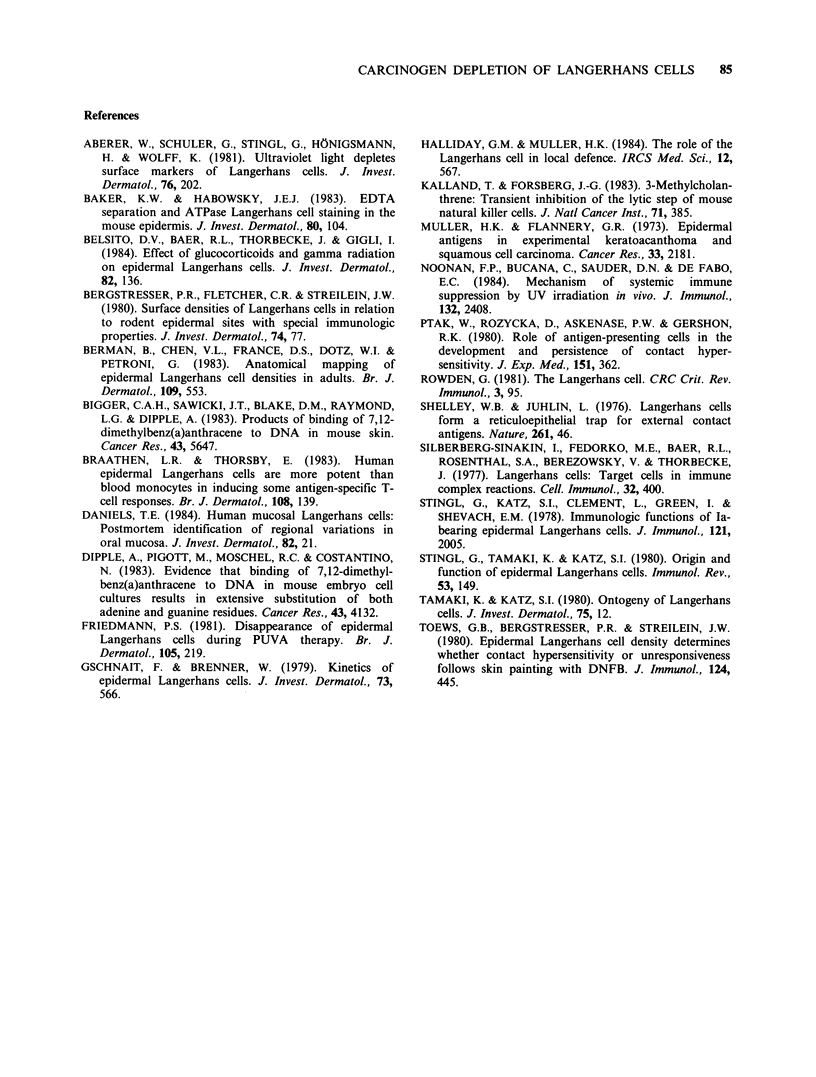# Carcinogen-induced depletion of cutaneous Langerhans cells.

**DOI:** 10.1038/bjc.1985.152

**Published:** 1985-07

**Authors:** H. K. Muller, G. M. Halliday, B. A. Knight

## Abstract

The chemical carcinogen 7,12-dimethylbenz (a) anthracene (DMBA) is a potent carcinogen which, when applied to the skin of BALB/c mice weekly for 7-8 weeks, causes the induction of macroscopically visible skin tumours. We report that DMBA also depletes Langerhans cells (LC) from treated skin; the number of cutaneous LC is reduced by nearly 50% 3 days after the first application of DMBA, and continues to decrease upon further treatment. After 7-8 weeks of DMBA application, while tumours are becoming macroscopically visible, there is a considerably lower LC density in treated skin. Upon cessation of the DMBA treatment, the LC repopulate the skin, returning to control values within 55-64 days. During this repopulation of the skin by LC, the tumours begin to decrease in size. Since LC function as local cutaneous antigen-presenting cells, and are responsible for initiation of an immune response against antigens in the skin, their depletion during tumour induction may allow DMBA-transformed cells to circumvent the immune system and form tumours. Their reappearance associated with tumour regression suggests that the LC are involved in an immune response against the tumours.


*Br. J. Cancer* (1985), **52**, 81–85

### Carcinogen-induced depletion of cutaneous Langerhans cells

H.K. Muller, G.M. Halliday & B.A. Knight

*Department of Pathology, University of Tasmania, G.P.O. Box 252C, Hobart, 7001, Australia.*

**Summary** The chemical carcinogen 7,12-dimethylbenz (a) anthracene (DMBA) is a potent carcinogen which, when applied to the skin of BALB/c mice weekly for 7–8 weeks, causes the induction of macroscopically visible skin tumours. We report that DMBA also depletes Langerhans cells (LC) from treated skin; the number of cutaneous LC is reduced by nearly 50% 3 days after the first application of DMBA, and continues to decrease upon further treatment. After 7–8 weeks of DMBA application, while tumours are becoming macroscopically visible, there is a considerably lower LC density in treated skin. Upon cessation of the DMBA treatment, the LC repopulate the skin, returning to control values within 55–64 days. During this repopulation of the skin by LC, the tumours begin to decrease in size. Since LC function as local cutaneous antigen-presenting cells, and are responsible for initiation of an immune response against antigens in the skin, their depletion during tumour induction may allow DMBA-transformed cells to circumvent the immune system and form tumours. Their reappearance associated with tumour regression suggests that the LC are involved in an immune response against the tumours.

Langerhans cells (LC) are the initial cells involved in a local immune response against antigens in the skin, where they function as antigen-presenting cells (Halliday & Muller, 1984). They are bone marrow derived dendritic cells (Stingl *et al.*, 1980) which bind antigen (Shelley & Juhlin, 1976) and transport it to the local lymph nodes (Silberberg-Sinakin *et al.*, 1977) for presentation to T cells, resulting in a specific immune response against the antigen.

LC have been shown to be necessary for initiation of immune responses both *in vivo* and *in vitro*. *In vivo* studies of mouse skin depleted of LC by ultraviolet (UV) light irradiation have shown that LC are necessary for the induction of contact sensitivity (Toews *et al.*, 1980). In these experiments skin depleted of LC by UV-light could not support initiation of contact sensitivity, although contact sensitivity could be initiated via untreated skin. Thus local depletion of LC abolished the potential for an immune response against antigens in that locality, while a normal immune response could be initiated against antigens in other sites. *In vitro* studies using monocyte-depleted lymphocytes cultured with epidermal LC and antigen have formally proven that LC are able to present antigen to lymphocytes, initiating a specific immune response (Stingl *et al.*, 1978; Braathen & Thorsby, 1983).

Since LC are essential for an immune response to develop against antigens in the skin, the possible role of LC in the initiation of an immune response against cutaneous neoplastic cells is of considerable importance. We therefore investigated the effects of the chemical carcinogen 7,12-dimethylbenz (a) anthracene (DMBA) on LC numbers and morphology as assessed by adenosine triphosphatase (ATPase) staining during the induction and growth of DMBA-induced skin tumours. DMBA caused depletion of LC from skin surrounding the tumours, and the LC reappeared as the tumours ceased growing. The time scale of reappearance of LC suggests they were being repopulated from bone-marrow derived precursors.

Correspondence: H.K. Muller.
Received 2 January 1985; and in revised form 15 March 1985.

### Materials and methods

### *Carcinogen treatment of mice*

Male BALB/c mice were obtained from Walter and Eliza Hall Institute, Melbourne; from 8–9 weeks of age they were treated with 7,12-dimethylbenz (a) anthracene (DMBA, Sigma, USA, Lot 24F-0052) as described by Muller & Flannery (1973). The dorsal trunk skin of the mice was shaved with electric clippers and painted with 1% DMBA dissolved in equal volumes of lanoline (Sigma, USA) and liquid paraffin (Supply and Tender, Hobart); control mice received solvent alone.

To determine the early effects of DMBA on LC before tumours became apparent, groups of 4 mice were treated with DMBA. The first application of DMBA was designated as being on day 0, with weekly applications for up to 4 weeks; mice being killed 3 or 7 days following the last treatment. To determine the LC density in DMBA-treated skin during growth of DMBA-induced tumours, the DMBA was applied weekly for 7–8 weeks, the final application of DMBA was designated as being on day 0.

© The Macmillan Press Ltd., 1985

*Preparation of epidermal sheets*

At various times after the final application of DMBA, non-tumour bearing DMBA-painted skin was removed for assessment of LC numbers. At least one control mouse was included with each group of DMBA-treated mice. Skin was removed from the same site for control and test mice. The mice were killed by cervical dislocation, and prior to surgical removal the dorsal trunk skin was shaved, depilated (Veet, Reckitt & Colman Ltd., UK), and rinsed of depilant with tap water. Epidermal sheets were prepared by a method modified from that of Baker & Habowsky (1983). The skin was divided into 5 mm squares using a perspex template and subcutaneous fat was removed. The skin was incubated overnight at room temperature in PBS (pH 7.3) containing 20 mM ethylenediaminetetraacetic acid (EDTA) and 0.001% trypsin (Boehringer, W. Germany, Lot 1313157). Following incubation, the skin was submerged in PBS containing 1 mM $MgCl_2$ ($PBS/Mg^{++}$), and the stratum corneum and dermal layers were gently removed from the epidermis using fine forceps and a dissecting microscope.

*ATPase staining of LC*

Epidermal sheets were stained for the enzyme adenosine triphosphatase (ATPase) by a method adapted from those of Baker & Habowsky (1983) and Daniels (1984). Epidermal sheets were washed in $PBS/Mg^{++}$; fixed in cacodylate-buffered 2% formaldehyde (pH 7.3) for 20 min at 4°C; washed with 3 changes of saline over 10 min; stained for 1 h at 37°C with 21 mM trizma-maleate buffer (pH 7.3, Sigma, USA, Lot 61C-5130) containing 0.36 mM disodium adenosine triphosphate (ATP, vanadium free, Sigma, USA, Lot 91F-7165), 10.37 mM $MgSO_4$, 2.2 mM $Pb(NO_3)_2$, and 0.25 M sucrose; washed with 2 changes of saline over 5 min; developed with 2 drops of 20% ammonium sulfide in 50 ml distilled water for 5 min at room temperature; and washed with 2 changes of saline over 5 min. The sheets were then counterstained with 2% methyl green (Schmid and Co., W. Germany, Lot 11105) in distilled water for 5 min, washed with 3 changes of saline over 10 min, and mounted on glass slides, with the dermal surface down, in glycerin: PBS (9:1, v:v).

*Enumeration of LC in epidermal sheets*

For each mouse the number of LC in the dorsal trunk skin were enumerated microscopically in 4 replicate epidermal sheets, the total area being 2.5 mm². To correct for stretching during preparation the area of each sheet was measured using a Bioquant II basic measuring program volume 185 (r & m biometrics, USA) for an Apple IIe computer.

*Histological identification of tumours*

DMBA-induced tumours were surgically excised, fixed overnight in 10% formalin in PBS (pH 7.3), and vacuum embedded in wax. Vertical sections were stained with haematoxylin and eosin for histological examination.

*Statistical analysis*

LC densities from DMBA-treated and control mice were compared using an unpaired Wilcoxon ranked sum test.

### Results

*Effect of DMBA treatment on LC density*

The early effects of DMBA treatment on LC density in mouse skin are shown in Figure 1. Control mice had a mean LC density of 420 $mm^{-2}$, while 3 days after the first application of DMBA this was significantly reduced to 226 $mm^{-2}$ ($P<0.05$). The number of LC continued to decrease, and by 7 days following the first application of DMBA was 189 $mm^{-2}$, which is significantly lower than the control values ($P<0.05$). Subsequent applications of DMBA further decreased the LC densities to 118 $mm^{-2}$ 7

**Figure 1** Langerhans cell numbers in mouse skin following weekly treatments with DMBA. Control mice were treated with solvent alone. Each point represents the average count of 4 replicate epidermal sheets from one mouse.

days after 2 weekly applications; 97 $mm^{-2}$ 7 days after 3 weekly applications, and 71 $mm^{-2}$ 7 days after 4 weekly applications, all of which are significantly lower than the control values ($P<0.05$). Following 8 weekly applications of DMBA no detectable LC were observed in 2 mice (Figure 2).

*LC density during growth of DMBA-induced tumours*

LC densities at various times following cessation of 7–8 weekly applications of DMBA are shown in Figure 2, the means for 10 day periods are shown in Table I. Tumours were becoming visible macroscopically during this time period (Table I). All cutaneous lesions had appeared by day 24, with an average of 3 per mouse and a range of 1 to 8. The tumours showed a spectrum of disordered cell growth ranging from squamous papillomas and keratoacanthomas to a few squamous cell carcinomas. The remainder of the lesions were identified as squamous keratoses. The tumours commenced to reduce in size by day 25, with 48% regressing by days 55–64 (Table I), indicating reduced tumour growth with time.

During the first 14 days following the last application of DMBA the number of LC was depleted by ~75%, and during this time 94% of the tumours became macroscopically visible. The LC density then linearly increased towards control values, remaining significantly depleted even at 45–54 days, but returning to control values by 55–64 days following the DMBA treatment. As the LC returned to control values the tumours stopped growing and began to decrease in size. The LC which initially reappeared in the epidermis had short, thickened dendrites; after 30–40 days typical interdigitating LC were present with similar morphology to control LC.

**Figure 2** Langerhans cell numbers in mouse skin which had been painted weekly with DMBA or solvent (control) for 7–8 weeks; the final application was designated Day 0. Each point represents the average count of 4 replicate epidermal sheets from one mouse.

**Table I** Tumour growth pattern and Langerhans cell numbers in mouse skin painted weekly with DMBA for 7–8 weeks

| *Time period since last application of DMBA (days)* | *Percentage of total tumours induced* | *Percentage of total tumours decreasing in size* | *Mean number of Langerhans cells mm$^{-2}$* |
|---|---|---|---|
| 0–4 | 53 | 0 | n.d. |
| 5–14 | 41 | 0 | 101 ($n=4$)[a] |
| 15–24 | 6 | 0 | n.d. |
| 25–34 | 0 | 7 | 339 ($n=6$)[a] |
| 35–44 | 0 | 28 | 223 ($n=4$)[a] |
| 45–54 | 0 | 44 | 332 ($n=4$)[a] |
| 55–64 | 0 | 48 | 385 ($n=4$)[b] |
| Control mice[c] | | | 425 ($n=10$) |

$n$ = number of mice.
n.d. = not done.
[a]Significance of difference from control $P<0.05$ (Wilcoxon rank sum test).
[b]Not significantly different from control (Wilcoxon rank sum test).
[c]Control mice were painted with solvent alone.

### Discussion

We have shown that the chemical carcinogen DMBA causes a rapid depletion of the local antigen-presenting LC during tumour induction, and that these cells reappear as the tumours stop growing and commence to decrease in size. This suggests that in the skin, the LC may have a role in protection against skin tumours, and that a defect in local antigen presentation may be required for a transformed cell to escape the immune system and multiply into a tumour.

LC were identified in epidermal sheets by staining for the plasma membrane bound enzyme ATPase which in the epidermis specifically stains LC (Rowden, 1981). Epidermal sheets were prepared by modification of a procedure which used incubation in EDTA at 37°C for 2.5 h to enable separation of dermis from epidermis (Baker & Habowsky, 1983). We found that inclusion of low concentration trypsin (0.001%), and incubation at room temperature overnight improved the LC morphology and ATPase staining, producing epidermal sheets in which the LC were observed to link with each other via their dendrites, allowing them to be more accurately quantitated. Using this procedure control BALB/c mice were found to have LC densities ranging from 340 to 690 $mm^{-2}$. Although other mouse strains have been reported with higher dorsal trunk epidermal LC densities e.g. 760 $mm^{-2}$ for C57BL mice and 880 $mm^{-2}$ for A/Jax mice (Bergstresser *et al.*, 1980), reported mean LC densities in normal epidermis vary considerably; in the case of human anterior forearm skin from 458 $mm^{-2}$ (Berman *et al.*, 1983) to 720 $mm^{-2}$ (Friedmann, 1981).

It is possible that identification of LC by the surface membrane component ATPase may detect modulation of the enzyme rather than disappearance of the cell, however it is not practical to count LC densities by electron microscopy, and any disruption of the plasma membrane which results in loss of ATPase should render the cells non-functional and alter their antigen trapping and presentation capabilities. Following exposure of mouse skin to UV-light, Aberer *et al.* (1981) found LC to be present by electron microscopy, although they were damaged and lacking in ATPase. In contrast Noonan *et al.* (1984) reported UV-light to deplete LC on both electron microscopy and ATPase staining criteria.

Glucocorticoid-mediated loss of ATPase from the LC membrane has been observed to require 7 days for the ATPase to be re-expressed on the LC membrane (Belsito *et al.*, 1984). In our experiments, the DMBA-treated LC-ATPase did not return to control values until 55–64 days following cessation of DMBA treatment. Considering this long recovery time, it is likely that DMBA depletes LC from the epidermis, and that the ATPase-positive cells which reappear in the epidermis following treatment are LC repopulating the epidermis rather than the original population re-expressing lost surface membrane ATPase. LC may repopulate the epidermis either from bone marrow precursors or from mitosis of residual LC. The time period required for the ATPase-positive cells to repopulate the epidermis is similar to the time required for donor bone marrow precursors to replace epidermal LC in chimeric mice (Tamaki & Katz, 1980). Repeated stripping of skin with adhesive tape, and dinitrochlorobenzene, both increase tritiated thymidine uptake in guinea pig LC, and although this may be due to proliferation of LC, it could also be due to increased DNA turnover during recovery of damaged LC (Gschnait & Brenner, 1979), particularly as mitosis of LC has only been observed on a few occasions (Rowden, 1981). Thus we concluded that the majority of the repopulated LC probably arose from bone-marrow precursors rather than from mitosis of residual LC. These experiments do not determine whether DMBA destroys the LC or causes it to leave the epidermis.

DMBA is known to depress natural killer cell activity (Kalland & Forsberg, 1983), thus inhibiting an immune effector mechanism against the tumour. DMBA has also been shown to react with deoxyadenosine in the DNA of mouse skin, indicating that its tumour inducing activity may be caused by a direct effect on DNA (Bigger *et al.*, 1983; Dipple *et al.*, 1983). However, our results suggest that as well as acting as a tumour inducer, DMBA also impairs immune surveillance by depleting epidermal antigen-presenting cells. This carcinogen-induced loss of LC may thus eliminate the potential for an immune response to develop against the transformed squamous cells, allowing these cells to proliferate.

It is also possible that the depletion of LC could result in the development of tumour-specific immunosuppression. Ptak *et al.* (1980) found that antigen coupled to epidermal cells induced immunity when injected intravenously, whereas antigen coupled to peritoneal exudate cells induced specific immunological unresponsiveness. It is possible that in the absence of LC, tumour antigens may filter through the skin and be presented to lymphocytes by monocytes, resulting in unresponsiveness. Further support for this comes from experiments by Toews *et al.* (1980), who reported that antigen applied to skin depleted of LC by UV-light resulted in specific unresponsiveness. However, Noonan *et al.* (1984), by using different wavelengths of UV-light claim that the UV-induced loss of LC is not responsible for the UV-induced systemic suppression; this requires further investigation.

The reduction in tumour size observed as the LC migrated back into the epidermis also suggests that the LC may be involved in an immune response against the tumours. However the mechanism of tumour regression has not been investigated, and its relationship with LC repopulation may be coincidental.

Finally, whether this DMBA-induced depletion of antigen-presenting cells is a general phenomenon associated with the induction of all tumours will only be apparent when the effects of other carcinogens are examined on other antigen-presenting cells in different regions of the body.

This study was supported by grants from the National Health and Medical Research Council of Australia and the Tasmanian Cancer Council. We than Miss N. Hummerstone and Mr T. Van Galen for technical assistance.

**References**

ABERER, W., SCHULER, G., STINGL, G., HÖNIGSMANN, H. & WOLFF, K. (1981). Ultraviolet light depletes surface markers of Langerhans cells. *J. Invest. Dermatol.*, **76**, 202.

BAKER, K.W. & HABOWSKY, J.E.J. (1983). EDTA separation and ATPase Langerhans cell staining in the mouse epidermis. *J. Invest. Dermatol.*, **80**, 104.

BELSITO, D.V., BAER, R.L., THORBECKE, J. & GIGLI, I. (1984). Effect of glucocorticoids and gamma radiation on epidermal Langerhans cells. *J. Invest. Dermatol.*, **82**, 136.

BERGSTRESSER, P.R., FLETCHER, C.R. & STREILEIN, J.W. (1980). Surface densities of Langerhans cells in relation to rodent epidermal sites with special immunologic properties. *J. Invest. Dermatol.*, **74**, 77.

BERMAN, B., CHEN, V.L., FRANCE, D.S., DOTZ, W.I. & PETRONI, G. (1983). Anatomical mapping of epidermal Langerhans cell densities in adults. *Br. J. Dermatol.*, **109**, 553.

BIGGER, C.A.H., SAWICKI, J.T., BLAKE, D.M., RAYMOND, L.G. & DIPPLE, A. (1983). Products of binding of 7,12-dimethylbenz(a)anthracene to DNA in mouse skin. *Cancer Res.*, **43**, 5647.

BRAATHEN, L.R. & THORSBY, E. (1983). Human epidermal Langerhans cells are more potent than blood monocytes in inducing some antigen-specific T-cell responses. *Br. J. Dermatol.*, **108**, 139.

DANIELS, T.E. (1984). Human mucosal Langerhans cells: Postmortem identification of regional variations in oral mucosa. *J. Invest. Dermatol.*, **82**, 21.

DIPPLE, A., PIGOTT, M., MOSCHEL, R.C. & COSTANTINO, N. (1983). Evidence that binding of 7,12-dimethylbenz(a)anthracene to DNA in mouse embryo cell cultures results in extensive substitution of both adenine and guanine residues. *Cancer Res.*, **43**, 4132.

FRIEDMANN, P.S. (1981). Disappearance of epidermal Langerhans cells during PUVA therapy. *Br. J. Dermatol.*, **105**, 219.

GSCHNAIT, F. & BRENNER, W. (1979). Kinetics of epidermal Langerhans cells. *J. Invest. Dermatol.*, **73**, 566.

HALLIDAY, G.M. & MULLER, H.K. (1984). The role of the Langerhans cell in local defence. *IRCS Med. Sci.*, **12**, 567.

KALLAND, T. & FORSBERG, J.-G. (1983). 3-Methylcholanthrene: Transient inhibition of the lytic step of mouse natural killer cells. *J. Natl Cancer Inst.*, **71**, 385.

MULLER, H.K. & FLANNERY, G.R. (1973). Epidermal antigens in experimental keratoacanthoma and squamous cell carcinoma. *Cancer Res.*, **33**, 2181.

NOONAN, F.P., BUCANA, C., SAUDER, D.N. & DE FABO, E.C. (1984). Mechanism of systemic immune suppression by UV irradiation *in vivo*. *J. Immunol.*, **132**, 2408.

PTAK, W., ROZYCKA, D., ASKENASE, P.W. & GERSHON, R.K. (1980). Role of antigen-presenting cells in the development and persistence of contact hypersensitivity. *J. Exp. Med.*, **151**, 362.

ROWDEN, G. (1981). The Langerhans cell. *CRC Crit. Rev. Immunol.*, **3**, 95.

SHELLEY, W.B. & JUHLIN, L. (1976). Langerhans cells form a reticuloepithelial trap for external contact antigens. *Nature*, **261**, 46.

SILBERBERG-SINAKIN, I., FEDORKO, M.E., BAER, R.L., ROSENTHAL, S.A., BEREZOWSKY, V. & THORBECKE, J. (1977). Langerhans cells: Target cells in immune complex reactions. *Cell. Immunol.*, **32**, 400.

STINGL, G., KATZ, S.I., CLEMENT, L., GREEN, I. & SHEVACH, E.M. (1978). Immunologic functions of Ia-bearing epidermal Langerhans cells. *J. Immunol.*, **121**, 2005.

STINGL, G., TAMAKI, K. & KATZ, S.I. (1980). Origin and function of epidermal Langerhans cells. *Immunol. Rev.*, **53**, 149.

TAMAKI, K. & KATZ, S.I. (1980). Ontogeny of Langerhans cells. *J. Invest. Dermatol.*, **75**, 12.

TOEWS, G.B., BERGSTRESSER, P.R. & STREILEIN, J.W. (1980). Epidermal Langerhans cell density determines whether contact hypersensitivity or unresponsiveness follows skin painting with DNFB. *J. Immunol.*, **124**, 445.